# The relationship and predictive value of dementia and frailty for mortality in patients with surgically managed hip fractures

**DOI:** 10.1007/s00068-023-02356-z

**Published:** 2023-09-01

**Authors:** Ioannis Ioannidis, Maximilian Peter Forssten, Ahmad Mohammad Ismail, Yang Cao, Lakshika Tennakoon, David A. Spain, Shahin Mohseni

**Affiliations:** 1 Department of Orthopedic Surgery, Orebro University Hospital, Orebro, Sweden; 2 School of Medical Sciences, Orebro University, Orebro, Sweden; 3 Clinical Epidemiology and Biostatistics, School of Medical Sciences, Faculty of Medicine and Health, Orebro University, Orebro, Sweden; 4 Department of Surgery, Section of Trauma and Acute Care Surgery, Stanford University School of Medicine, Stanford, CA USA; 5 Division of Trauma, Critical Care & Acute Care Surgery, Department of Surgery, Sheik Shakhbot Medical City Mayo Clinic, Abu Dhabi, United Arab Emirates

**Keywords:** Hip fracture, Frailty, Dementia, In-hospital mortality, Prediction

## Abstract

**Background:**

Both dementia and frailty have been associated with worse outcomes in patients with hip fractures. However, the interrelation and predictive value of these two entities has yet to be clarified. The current study aimed to investigate the predictive relationship between dementia, frailty, and in-hospital mortality after hip fracture surgery.

**Methods:**

All patients registered in the 2019 National Inpatient Sample Database who were 50 years or older and underwent emergency hip fracture surgery following a traumatic fall were eligible for inclusion. Logistic regression (LR) models were constructed with in-hospital mortality as the response variables. One model was constructed including markers of frailty and one model was constructed excluding markers of frailty [Orthopedic Frailty Score (OFS) and weight loss]. The feature importance of all variables was determined using the permutation importance method. New LR models were then fitted using the top ten most important variables. The area under the receiver-operating characteristic curve (AUC) was used to compare the predictive ability of these models.

**Results:**

An estimated total of 216,395 patients were included. Dementia was the 7th most important variable for predicting in-hospital mortality. When the OFS and weight loss were included, they replaced dementia in importance. There was no significant difference in the predictive ability of the models when comparing the model that included markers of frailty [AUC for in-hospital mortality (95% CI) 0.79 (0.77–0.81)] with the model that excluded markers of frailty [AUC for in-hospital mortality (95% CI) 0.79 (0.77–0.80)].

**Conclusion:**

Dementia functions as a surrogate for frailty when predicting in-hospital mortality in hip fracture patients. This finding highlights the importance of early frailty screening for improvement of care pathways and discussions with patients and their families in regard to expected outcomes.

## Background

The high risk of mortality and morbidity after hip fracture surgery remains a healthcare concern, with reported post-operative mortality as high as 10% and 27% at 30-days and 1-year, respectively [1, 2]. Up to 20% of patients with hip fracture have a dementia diagnosis, [3], and it has been established that this group is disproportionally at risk for postoperative adverse outcomes [4, 5]. Due to the global increase in life expectancy with an aging population, it is anticipated there will be an increase in patients suffering from both hip fractures and dementia [6].

A recent large study using data from the national Swedish Hip Fracture registry found that dementia was a surrogate for frailty when predicting postoperative mortality in hip fracture patients. Furthermore, the presence of dementia in a patient without frailty did not appreciably contribute to the prediction of postoperative mortality [7]. This finding is important for resource allocations, perioperative care management, discussions in regards to expected outcomes with patients and their relatives, as well as in outcome research investigations in this patient population. Using the United States National Inpatient Sample (NIS) database, the authors endeavor to validate these findings. The objective was to investigate whether dementia serves the same purpose as frailty when predicting in-hospital mortality following a traumatic hip fracture. The hypothesis was that dementia would function as a surrogate for frailty when predicting mortality.

## Methods

The current study utilized data from the 2019 United States NIS, which is recognized as the largest all-payer inpatient database in the United States. The NIS is overseen by the Agency for Healthcare Research and Quality, and it surveys 20% of all hospitalizations in the country to ensure accurate national estimates for 97% of all inpatient hospitalizations in the United States through validated sampling algorithms with discharge and survey weight. [8]. To ensure adherence to rigorous scientific reporting standards, the study complied with the STrengthening the Reporting of OBservational studies in Epidemiology (STROBE) guidelines and principles of the Declaration of Helsinki [9]. Additionally, ethical approval for the study was obtained from the Swedish Ethical Review Authority (ref: 2022-03107-02).

The study included all adult patients 50 years or older who had undergone emergency hip fracture surgery following a traumatic fall and were managed surgically using internal fixation (open reduction internal fixation or intramedullary nailing) or arthroplasty (total hip arthroplasty or hemiarthroplasty). To mitigate heterogeneity in the dataset, patients with head, vascular, or truncal injuries were excluded. Patients missing data were also excluded in order to allow for a complete case analysis. The variables were identified through International Classification of Diseases 10th Revision (ICD-10) codes registered in the NIS [10]. The dataset included patient demographics, clinical characteristics, markers of frailty [Orthopedic Frailty Score (OFS) and weight loss], [11–14], as well as discharge disposition.

### Statistical analysis

In order to provide a concise summary of the study population, patients were divided into those who survived the hospital stay, and those who died in-hospital. Age was presented as a median and interquartile range as it was not normally distributed; differences between the groups were subsequently assessed using the Mann-Whitney U test. Categorical variables were summarized using counts and percentages. Differences between categorical variables were determined using the Chi-squared test or Fisher’s exact test. The primary outcome was in-hospital mortality.

A logistic regression (LR) models was fitted with in-hospital mortality as the response variable [7]. The predictors included age, sex, race/ethnicity, median household income, markers of frailty, type of fracture, type of surgery, comorbidities, and administered medications. The markers of frailty were the OFS and weight loss [11–14]. Comorbidities included dementia, hypertension without complications, hypertension with complications, ischemic heart disease, angina pectoris, previous myocardial infarction, congestive heart failure, valvular disease, peripheral vascular disorder, diabetes mellitus without complications, diabetes mellitus with complications, chronic kidney disease, cerebrovascular disease, paralysis, other neurological disorder, pulmonary circulation disorder, chronic pulmonary disease, chronic obstructive pulmonary disease, peptic ulcer disease, liver disease, coagulopathy, connective tissue disease including rheumatoid arthritis, connective tissue disease excluding rheumatoid arthritis, chronic blood loss anemia, deficiency anemia, fluid/electrolyte disorder, obesity, osteoporosis, osteoporosis with previous fracture, previous deep vein thrombosis, AIDS, local cancer, leukemia, lymphoma, metastatic cancer, depression, psychosis, current smoker, alcohol abuse, drug abuse. Administered medications included insulin, antithrombotic medications, anticoagulants, and corticosteroids.

Two models were fitted, one including all variables *except* for the markers of frailty, and another *including* the markers of frailty. The relative importance of all variables in each model was determined using the permutation importance (PI) method. [15]. The PI was determined by estimating the effect of omitting a specific variable on a predetermined value [1 - Area under the receiver-operating characteristic curve (AUC)]. The PI method masks the information of a variable during evaluation by replacing it with noise from other cases. To account for the uncertainty related to the use of permutations, this process was repeated 10 times for each model. The relative importance of each variable in the model was then presented as the average increase in 1-AUC relative to the AUC in a model including all variables without masking.

As a final step, two new LR models were fitted using the top ten most important variables according to their relative importance. [7]. The AUC for each model was calculated and used to compare the predictive ability of the two models, one *with* and one *without* markers of frailty, for each outcome. This was done to determine whether the markers of frailty improved the predictive ability of the models, or whether they served as a replacement for dementia.

Statistical significance was defined as a two-sided p-value <0.05. The analyses were performed using the statistical programming language R (R Foundation for Statistical Computing, Vienna, Austria), with the *tidyverse*, *DALEX*, *haven*, *survey*, *cowplot*, *pROC*, and *parallel* packages [16].

## Results

This analysis included an estimated total of 216,395 patients; 1.3% (N = 2,820) died in the hospital. Those that died in the hospital were generally older [86 vs 81 years old, p <0.001] and a larger proportion was male [44.9% vs 30.5%, p <0.001]. There was no significant difference in race/ethnicity and median household income by zip code. Patients who died in the hospital were much more likely to be classified as frail according to their OFS [OFS ≥2: 34.9% vs 13.8%, p <0.001], suffer from unintentional weight loss [16.1% vs 7.6%, p <0.001], and dementia [34.6% vs 25.8%, p <0.001]. Those who died were also less likely to have suffered a cervical hip fracture [37.6% vs 47.4%, p <0.001]; however, there was no difference in the overall use of internal fixation and arthroplasty to manage the fractures. A generally higher comorbidity burden was also observed among patients who died in the hospital (Table 1).

**Table 1:** Demographics and comorbidities of hip fracture patients

	Survived (N = 213,575)	In-hospital death (N = 2,820)	P-value*
Age, median [IQR]	81 [72.0–88.0]	86 [79.0–90.0]	<0.001
Sex, n (%)			<0.001
Male	65,130 (30.5)	1265 (44.9)	
Female	148,445 (69.5)	1555 (55.1)	
Race/Ethnicity, n (%)			0.535
White	184,445 (86.4)	2475 (87.8)	
Black	8730 (4.1)	105 (3.7)	
Hispanic	11,550 (5.4)	140 (5.0)	
Asian or Pacific Islander	3780 (1.8)	50 (1.8)	
American Indian	865 (0.4)	20 (0.7)	
Other	4205 (2.0)	30 (1.1)	
Median household income by zip code, n (%)			0.456
0–25th percentile	55,645 (26.1)	810 (28.7)	
26–50th percentile	55,250 (25.9)	725 (25.7)	
51–75th percentile	54,765 (25.6)	710 (25.2)	
76–100th percentile	47,915 (22.4)	575 (20.4)	
OFS, n (%)			<0.001
0	101,765 (47.6)	525 (18.6)	
1	82,395 (38.6)	1310 (46.5)	
2	26,080 (12.2)	850 (30.1)	
3	3110 (1.5)	115 (4.1)	
4	225 (0.1)	20 (0.7)	
Weight loss, n (%)	16,205 (7.6)	455 (16.1)	<0.001
Dementia, n (%)	55,115 (25.8)	975 (34.6)	<0.001
Type of fracture, n (%)			<0.001
Cervical	101,315 (47.4)	1060 (37.6)	
Basicervical	3465 (1.6)	30 (1.1)	
Pertrochanteric	99,900 (46.8)	1600 (56.7)	
Subtrochanteric	8895 (4.2)	130 (4.6)	
Type of surgery, n (%)			0.132
Internal fixation	134,670 (63.1)	1865 (66.1)	
Arthroplasty	78,905 (36.9)	955 (33.9)	
Hypertension without complications, n (%)	101,500 (47.5)	700 (24.8)	<0.001
Hypertension with complications, n (%)	60,150 (28.2)	1555 (55.1)	<0.001
Ischemic heart disease, n (%)	53,925 (25.2)	1290 (45.7)	<0.001
Angina pectoris, n (%)	225 (0.1)	0 (0.0)	0.440
Previous myocardial infarction, n (%)	3120 (1.5)	355 (12.6)	<0.001
Congestive heart failure, n (%)	33,820 (15.8)	1215 (43.1)	<0.001
Valvular disease, n (%)	13,170 (6.2)	160 (5.7)	0.629
Peripheral vascular disorder, n (%)	10,160 (4.8)	105 (3.7)	0.251
Diabetes mellitus without complications, n (%)	19,370 (9.1)	145 (5.1)	0.001
Diabetes mellitus with complications, n (%)	25,700 (12.0)	285 (10.1)	0.162
Use of insulin, n (%)	14,670 (6.9)	185 (6.6)	0.773
Chronic kidney disease, n (%)	44,895 (21.0)	1060 (37.6)	<0.001
Cerebrovascular disease, n (%)	13,355 (6.3)	300 (10.6)	<0.001
Paralysis, n (%)	7435 (3.5)	100 (3.5)	0.933
Other neurological disorder, n (%)	32,125 (15.0)	275 (9.8)	<0.001
Pulmonary circulation disorder, n (%)	1270 (0.6)	100 (3.5)	<0.001
Chronic pulmonary disease, n (%)	37,995 (17.8)	440 (15.6)	0.177
COPD, n (%)	5265 (2.5)	90 (3.2)	0.270
Peptic ulcer disease, n (%)	645 (0.3)	30 (1.1)	0.001
Liver disease, n (%)	4060 (1.9)	50 (1.8)	0.825
Coagulopathy, n (%)	11,640 (5.5)	160 (5.7)	0.816
Use of antithrombotic medication, n (%)	49,945 (23.4)	560 (19.9)	0.049
Use of anticoagulant, n (%)	28,850 (13.5)	450 (16.0)	0.091
Use of corticosteroid, n (%)	3305 (1.5)	45 (1.6)	0.926
Connective tissue disease including rheumatoid arthritis, n (%)	7005 (3.3)	30 (1.1)	0.003
Connective tissue disease excluding rheumatoid arthritis, n (%)	6945 (3.3)	35 (1.2)	0.007
Chronic blood loss anemia, n (%)	2450 (1.1)	10 (0.4)	0.078
Deficiency anemia, n (%)	31,380 (14.7)	245 (8.7)	<0.001
Fluid/electrolyte disorder, n (%)	52,725 (24.7)	1,240 (44.0)	<0.001
Obese, n (%)	9480 (4.4)	50 (1.8)	0.002
Osteoporosis, n (%)	33,105 (15.5)	265 (9.4)	<0.001
Osteoporosis with previous fracture, n (%)	2215 (1.0)	30 (1.1)	0.950
Previous deep vein thrombosis, n (%)	7930 (3.7)	120 (4.3)	0.499
AIDS, n (%)	160 (0.1)	5 (0.2)	0.381
Local cancer, n (%)	3865 (1.8)	65 (2.3)	0.382
Leukemia, n (%)	1335 (0.6)	10 (0.4)	0.417
Lymphoma, n (%)	1545 (0.7)	10 (0.4)	0.303
Metastatic cancer, n (%)	2285 (1.1)	60 (2.1)	0.016
Depression, n (%)	23,875 (11.2)	125 (4.4)	<0.001
Psychosis, n (%)	5355 (2.5)	45 (1.6)	0.168
Current smoker, n (%)	1315 (0.6)	15 (0.5)	0.800
Alcohol abuse, n (%)	6915 (3.2)	40 (1.4)	0.015
Drug abuse, n (%)	2515 (1.2)	25 (0.9)	0.524
Length of stay, median [IQR]	4 [3.0–6.0]	5 [3.0–9.0]	0.055

*OFS* Orthopedic Frailty Score, *COPD* chronic obstructive pulmonary disease

**Fig. 1:**
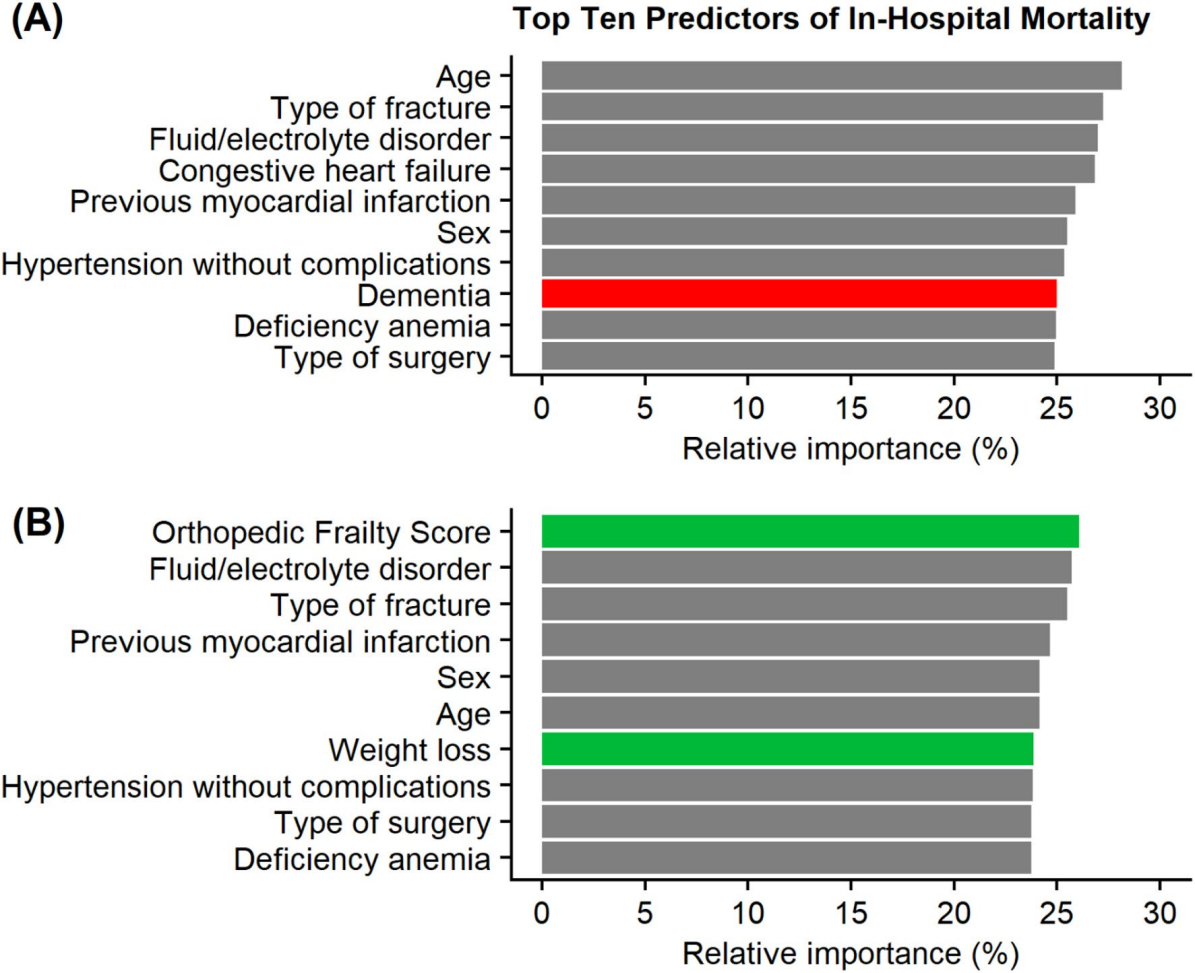
Top ten predictors for in-hospital mortality based on the models excluding (**A**) and including (**B**) markers of frailty

When ranking the relative importance of variables for predicting in-hospital mortality, *excluding* markers of frailty, dementia was the 7th most important variable. When markers of frailty were *included,* dementia was replaced in rank by the OFS and weight loss. The top ten most important variables for predicting in-hospital mortality and their relative ranks are presented in Figure 1. There was no significant difference in the predictive ability of the LR models built using the top ten most important variables when comparing those that *included* [AUC (95% CI): 0.79 (0.77–0.81)] and *excluded* [AUC (95% CI): 0.79 (0.77–0.80)] markers of frailty. When all 52 variables were used to build the LR models, the predictive ability of the model remained relatively unchanged [AUC (95% CI): 0.81 (0.79–0.83)] (Table 2).

**Table 2:** Predictive ability of logistic regression models built using the top ten most important variables

Outcome	AUC for model excluding frailty (95% CI)	AUC for model including frailty (95% CI)	AUC for model using all variables (95% CI)
In-hospital mortality	0.79 (0.77–0.80)	0.79 (0.77–0.81)	0.81 (0.79–0.83)

*AUC* Area under the receiver-operating characteristic curve, *CI* confidence interval

## Discussion

In the current analysis, dementia is replaced in predictive importance by the OFS and weight loss when predicting in-hospital mortality. Both of the model excluding and the model including markers of frailty performed at the same level when predicting in-hospital mortality. Dementia consequently appears to function as a surrogate for frailty when predicting in-hospital mortality.

These results are in accordance with a previous investigation utilizing the National Swedish Hip Fracture Register, which studied the role of dementia and frailty in predicting mortality up to 1 year postoperatively in patients with hip fractures. This study incorporated several markers of frailty, including functional status, institutionalization, walking ability, and the use of walking aids. Notably, the results demonstrated that these markers of frailty replaced dementia in terms of predictive importance at all investigated time points. This further indicates that the frailty measured by OFS and weight loss, as well as markers of frailty included in the previous study, adequately capture the concept of frailty. Finally, both the current models and those from the previous investigation, using a large Swedish cohort, for 30-day mortality exhibited an AUC around 0.8, demonstrating a robust predictive capability. Moreover, all models surpassed an AUC threshold of 0.7, signifying that a significant proportion of the important predictors of mortality were captured by the datasets [7, 17].

Given the high mortality rate observed in frail hip fracture patients, [11, 12, 18], and in particular those with dementia, [3], identifying potential avenues for mitigating this adverse outcome is essential. One potential approach is the orthogeriatric care model. This model emphasizes the collaborative efforts between orthopedic surgeons and geriatricians to provide specialized care that addresses the unique medical, cognitive, and functional challenges faced by this population. Studies have consistently demonstrated the advantages of the orthogeriatric care model in terms of improved outcomes and reduced healthcare resource utilization, cost, hospital stay, as well as a reduction in morbidity and mortality [19–24]. Another potential intervention worth considering is beta-blocker therapy, which has been proposed as a means to reduce the posttraumatic hyperadrenergic response. [25–28]. Previous investigations have found an association between preadmission beta-blocker therapy and a reduction in postoperative mortality in hip fracture patients with dementia, a reduction which has also been observed to be larger the more frail a patient was. [29, 30]. Nevertheless, future studies will be required to determine if there is any advantage to the initiation of beta-blocker therapy in hip fracture patients.

This investigation included almost 220,000 estimated patients with hip fractures from the NIS, the largest all-payer inpatient database in the United States, raising both the external and internal validity of the results [8]. By leveraging this dataset, a wide range of potential predictors could be included in the regression models. Furthermore, the study design limited the population to patients with surgically managed hip fractures without significant additional injuries, thus reducing the overall heterogeneity in the study population. However, it is important to acknowledge that the retrospective nature of the study poses certain limitations. Potential predictors that were not captured by the dataset, such as time to surgery, preoperative optimization, admission vitals, and ongoing pharmacotherapies, could not be included in the analysis. The analyses conducted in this study were also confined to the outcomes available within the dataset, thereby precluding assessments of post discharge mortality, functional status, or quality of life. While the analyses validated that dementia serves as a surrogate for frailty when predicting mortality in hip fracture patients, it is important to note that this does not necessarily mean that all patients with dementia are inherently frail. Nevertheless, there remains a significant correlation between dementia and frailty. It should also be emphasized that this study assumes that the OFS and weight loss are markers of frailty, as established by previous research; [11, 14, 18, 30]; however, it should be acknowledge that these variables indicate the presence of frailty rather than encompassing the concept of frailty itself. Finally, we were only able to establish the association between dementia, frailty and mortality for in-hospital mortality, whereas former studies used up to 1-year mortality [7, 11, 18, 30]. Despite the different timepoints for mortality, the results of the current investigation are consistent with previous studies, confirming the robust nature of these results.

## Conclusion

Dementia functions as a surrogate for frailty for predicting in-hospital mortality in patients who have undergone hip fracture surgery. This finding highlights the importance of early frailty screening for improvement of care pathways and discussion with patients and their families in regard to expected outcomes.

## Conflict of interest

The authors have no conflicts of interest to disclose.
